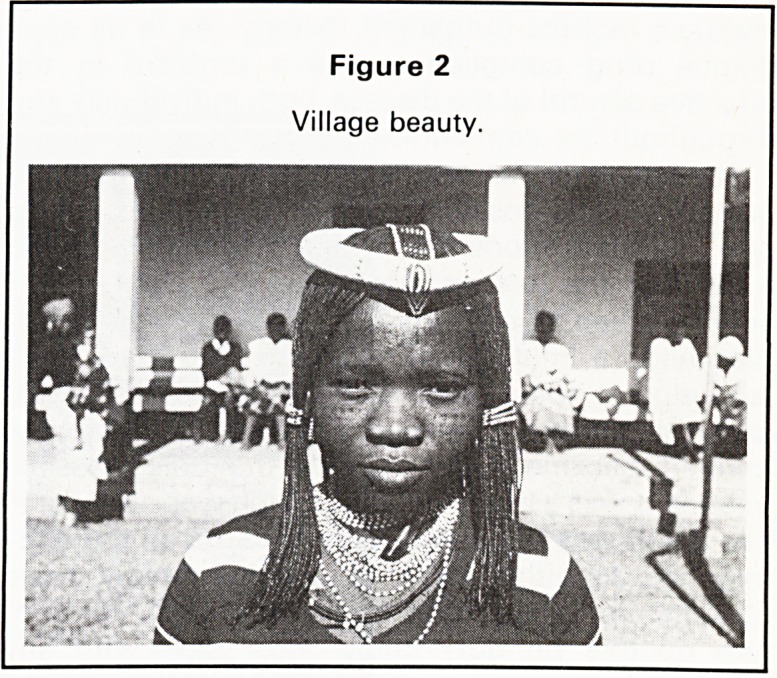# From Our Foreign Correspondent

**Published:** 1985-04

**Authors:** 


### From Our Foreign Correspondent

### Bangladesh – 1977 to 1984

My first view of Bangladesh was in 1977 when I joined the staff of the R.I.H.D. Hospital, classified as a Technical Assistant for the Overseas Development Ministry – it reverted to an Administration in the Foreign Commonwealth Ministry with the return of the Conservative Government in 1979. These appointments are financed by the O.D.A. and an orthopaedic surgeon can usually manage only a 4 to 5 weeks absence from a busy practice. As I am retired I have always spent 3 months and in all have spent 18 months in Bangladesh, five in the pleasant winter months but, in 1984 in the hot humid summer when, as in many other years, major flooding led to very many deaths from drowning, loss of crops and the evacuation of many homesteads.

Only in Sylhet in the extreme East and in the Hill Tracts behind Chittigong is there any high ground, the remainder of Bangladesh consists of the delta and the lower reaches of the three rivers, the Ganges, the Bramaputra and the Meghna. The area of Bangladesh is about 60 per cent of the United Kingdom, the population over 90,000,000 resulting in a density of population in the dry season three times that in this country. When the land is very largely flooded the population density is immensely greater and in the cities overpowering and consisting almost entirely of men and boys as the women in this Moslem country mostly remain indoors, even the shopping being done by the men. In the country all houses and farms are built on the highest available land and most are able to withstand the annual flooding; the roads are constructed above the level of the surrounding fields and river banks are raised; disatser overtakes the area when these banks are breached.

The annual flooding is an essential part of the annual agricultural cycle and produces the main rice crop which is planted by hand as individual seedlings (8″ tall) when the fields are covered with no more than 6″ of water. This main crop does not require irrigation and is harvested by December. Crops of rice are also grown on the banks of rivers as the water level falls and areas are left of suitable depth for progressive further planting. Relatively simple pumps operating from the river suffice for irrigation of these later crops. With so much warmth and sunlight three crops in a year are possible but only with irrigation schemes. On one side of the road one can see a virtual desert while on the other, deep well water pumps sustain a luxurious growth of rice and other crops. Finally, as the water rises in the monsoon season, deep water rice is planted, maintaining its head above water even when a depth of 6 feet is reached at the height of the flooding. These were the crops washed away in the 1984 floods.

**Figure 1**

A recently planted paddy field showing the individually planted seedlings. The homesteads and the roads are raised above the level in the fields.

Besides rice, which is the staple crop, wheat is growing in popularity as a winter grown cereal. Jute is still the crop earning most foreign currency but has suffered from the fact that at partition in 1947, most jute was grown in East Bengal or Bangladesh while the jute mills were mostly in Indian West Bengal. The competition of man made fibre continues to grow and adversely affect jute sales. Cane sugar, cotton, tobacco, mustard, vegetable and many fruits flourish on the rich silt washed down annually from the high ground in Northern India, Nepal and Tibet. The rains falling in Assam and North East India are the heaviest in the world and it is these rather than the actual rainfall in Bangladesh which cause the flooding in this vast delta. Tea only grows on the higher ground, notably in Sylhet.

The population of Dhaka is so great that in parts of the city the tricycle rickshaws form a solid mass from pavement to pavement and the cars, Japanese baby taxis (motor cycle three-wheelers) and buses can make very slow progress amongst them, despite continuous use of the horn. Elsewhere the buses and lorries act in a despotic manner – power, weight and a young driver. The tricycle rickshaws or trishaws, although troublesome for traffic movement, in numbers far exceeding the licenses issued, are essential to the community by giving employment for many and a cheap personal transport for the population. They are also used to transport goods, often with a passenger perched on top of the sacks, or behind the table he is succeeding in keeping balanced. Being tipped out of a trishaw is a not infrequent road accident.

Poverty and dense population leads to families with no homes save on a portion of public pavement and great areas of shacks with very limited facilities acting as homes to the innumerable poor. The railway station at night gives shelter to many sleeping in a solid mass on the floor, through which a narrow lane is maintained for late travellers, reminding one of similar scenes in all the tube stations in London during the World War II.

**Figure 2**

Craftsmanship and use of materials available locally. Scaffolding of bamboo and string for the tower on the new international airport at Dhaka.

Workmen squat down at various cross roads with their baskets and broad bladed hoes which act as spades, awaiting someone to hire their labour. Whole families are engaged in breaking up bricks to form aggregate on building sites – there is no stone in this delta. The owners of the heavy wooden carts which transport heavy loads also await someone to hire them and these men are the traction force. Yes, man is the commonest beast of burden in Bangladesh, the rickshaw puller, the men moving these heavy carts and those who pull the boats on the rivers with ropes coming from the top of the mast, thus avoiding entanglement with smaller craft lying inshore.

Buses and trains, the public transport, are always overladen with passengers, outside as well as within, the former sometimes with but slender holds. Although cows and calves wander the streets of Dhaka at will, ox and bullock carts are rare and forbidden on many roads. These cattle in the fields draw the wooden plough with its iron tip and it is the stronger water buffalo which is more often seen in the waterlogged paddy field. Horses are only seen in certain areas and the tractor only on the large estates, usually tea estates.

Politics always seem to be at boiling point and the Universities are torn by national politics with one party calling a strike and preventing students from other parties from attending lectures. In Rajshahi in 1984, a student strike at the Medical School had persisted for five months, the result of the Medical School Authorities refusing to remove the Dean at the students' request. Injury and death are frequent as a result of political fanaticism. The present political cycle appears to be a coup d'etat by an army officer, the creation of his personal political party and then he becomes the President, ratified at an appropriately timed referendum. At the moment, the contention that a free election cannot take place while Martial Law persists is matched by the equally sensible argument that a power vacuum would succeed it and be equally uncongenital to free elections. Political parties all tend to be based on personal allegience and rarely on ideology.

Bangladesh is a Moslem country and in the last three years it has been more restrictive on the consumption of alcohol, more prohibitive of proselytisation by any other religion and has changed from Sunday to Friday as the day off work. But there is no Sharia (Moslem Law) and Hindus comprise an appreciable percentage of the population in some areas, notably Mymensingh. One of the continuing population problems remain the Bihari, Moslems who moved into East Pakistan at the Partition in 1947, ran the railways and much of the civil service and police, sided with the West Pakistanis during the war of Independence (1971) so becoming the enemy remaining within the land when that war ended. Herded into camps and comprising some 700,000 at the time, the remainder still number 300,000 and await the opportunity to return "home" to Pakistan, a country they have never seen and from which they do not derive. Pakistan, apart from Bangladesh, is the only Moslem country in the Indian subcontinent and they did fight with the West Pakistanis in the war of Independence.

The artistic culture is much in evidence in the wealth of decoration on trishaws and trucks with exotic and colourful designs of flowers, animals, humans and even arctic scenes! The private car, baby taxi and bus are completely neglected in this respect – but there is clearly a very profitable industry in producing these decorative displays. The general poverty cramps many cultural expressions. The old capital city of Sonanguon, 12 miles from Dhaka dates from before the 13th century and has only recently had any money spent on its restoration and preservation. Old Dhaka has some most attractive buildings, two-stored with balcony and beautiful

**Figure 3**

Sonangaon—the old Capital City in this part of Bengal with a glimpse of an aspect of the past. Records go back to the 13th Century.

decoration, but everything is in such poor repair and in such desperate need of paint that the general view is not pleasing. The roads in Old Dhaka are so narrow that I gave up driving a car, first at night and more recently only penetrating rarely by day with a well informed Bangladesh navigator.

There is a fine new Museum opened in the last year to replace the decrepit museum near Old Dhaka. Their literary genius is recalled by the poet Tagore and by Sher-e-Bangla and no doubt continues to flourish today.

With such a tremendous income difference between many Europeans and the majority of the Bangladeshis there develops two cultures running alongside each other, only a few bridging the gap. A number of Bangladeshis are clearly in the upper financial bracket. Begging becomes inevitable and there is a natural feeling that most Europeans should pay substantially more and this all adds considerably to the strain of living in Bangladesh. The rich Bangladeshi, from centuries of experience, handles these situations with such aplomb and clearly the expectation of the rickshaw puller is far less. With each visit the European improves his performance but such discrepancies must always be painful.

### Orthopaedic and Medical aspects

The hospital – Rehabilitation Institute Hospital for the Disabled (RIHD) – has a history to explain its unwieldly title. Professor Ronald Garst and his wife, Marie, in February 1972 came over from India, where he had for nearly quarter of a century been Professor of Orthopaedic Surgery at the Ludhiane Christian Medical College in the Punjab. They offered help to the Government of this poor country, attempting to make a start as an independent nation after the very damaging war which had culminated in victory two months previously.

The backing of the Christophe Blinden Mission in West Germany enabled him to accept the proposed outpatient clinic and convert it into a busy orthopaedic hospital for 'Freedom Fighters' and other casualties of the war. Beds, autoclaves, basic radiographic and theatre equipment were imported and Bangladeshi staff recruited to augment some Indian colleagues who joined them. The Government had initially agreed to take over the hospital in twelve months and this actually took place fifteen months after the inception of the project. Professor Garst agreed to continue to run the hospital if allowed to continue and expand the orthopaedic surgery training programme. This teaching role always entails the use of the term "Institute" which consequently took its place in the title. The name is now often abbreviated to 'Pongo' or fracture hospital or even orthopaedic hospital and, since those days, it has moved to a 400 bedded four storeyed brick building, constructed from monies allocated by the Christophe Blinden Mission and the Bangladesh Government in equal amounts.

The move to this building occurred in March 1978 as I left at the end of my first term. Professor Garst with his remarkable drive, enthusiasm, life long knowledge of the Indian subcontinent, tact and diplomacy, built up the orthopaedic training programme. The staffing of this teaching unit, on which a population of 90,000,000 will depend for its orthopaedic surgical service, was supplemented by orthopaedic surgeons sent out by the Overseas Development Administration or Ministry from the United Kingdom and by American orthopaedic surgeons organised by "Orthopaedic Overseas" and assisted by CARE. In the United States all monies given to charity are exempted from tax so that these surgeons came over for 3–4 weeks and these were usually initial visits so that the special circumstances had to be learnt by each visiting surgeon. The surgeon's visit was financed from untaxed income and combining it with a later visit to Nepal or India, in which he was joined by his wife, proved an attractive proposition. They stayed less time in Bangladesh but gave their services without payment and those who returned in particular gave excellent service to Bangladesh and combined it with a holiday. The British surgeons were paid by O.D.A. and almost invariably came unaccompanied. There is not much for a wife to do in Dhaka after her husband leaves the abode *with the car*.

The orthopaedic surgical trainees have a daily conference and, after a lecture each morning of a six day week, are fully involved in the work and took a Diploma in 2 years or a Mastership in Orthopaedic Surgery in $3\frac{1}{2}$ years. They are posted out to one of the eight Medical Colleges with, in the early days, little experience after passing their exams. They often met with difficulties as they pioneered the new discipline in the departments of Surgery, manned by Professors of Surgery really practising surgery in general and with the omnipotence often felt by such surgeons viewing their widespread domain. Many of the pioneering young orthopaedic surgeons have done very well and carved out their domain in which their results have justified their existence as a very large branch of surgery in general. The scene recalled that in Britain fifty years ago.

Other young orthopaedic surgeons have gone to Saudi Arabia, Libya and formerly to Iran; I know of six young surgeons working at this time in these oil rich countries. They are earning good money which will enable them to have a car, buy a house or a plot of land as the Government house is only a "tied cottage" and must be relinquished on retirement. The Government does not frown on this practice, although attempting to control it, as it earns much needed foreign currency – these earnings by Bangladeshies in Saudi Arabia are a very important element in the Government's external budgeting. The young surgeons often appear to need the money and some experience of the outside world before they can settle down, but they write to me quoting my remarks that Bangladesh has given them their training and needs their services and assuring me that they will return. It is often 3–4 years before this happens and I am sure that Saudi Arabia attempts to delay their return. This period of working in these oil rich countries seems to be a reality in the scheme of things which one just has to accept – an unavoidable delay, but a returning surgeon settled to do his life work in his country.

The Mission funds enabled Professor Garst to do so much more than was possible on Government funding alone, he developed a school of Physiotherapy and Occupational Therapy in which the British V.S.O.s played the dominant part and I have a tremendous admiration for the work in so many fields of these Volunteers, usually young and spending two years abroad and 'willing to work at local pay only'. Orthotists and prosthetists were also trained and when these and the physiotherapists were posted to the Medical Schools outside Dhaka, they continued to be financed from Mission funds.

The R.I.H.D. suffered a severe loss when Professor Garst retired in 1981 and with this blow came a reduction in the funding from C.M.B. This ceased completely by 1985 but this is a reality of all external funding; it cannot continue indefinitely. As the Government did not actually build this hospital it does not always enthuse over maintenance, adding to the difficulties of the Bangladeshi Project Director of the hospital in the last 4 years.

The ideal tour for me has been four weeks teaching and being involved in the work of the wards, clinics and operating theatres, followed by the exam which is always the central focus for the visit. Following this one travels to two or three medical colleges, spending the inside of a week at each. The orthopaedic surgeon there is always well known to me and the visit is valuable to everyone in that it supports his Unit, gives opportunity for discussion, to share responsibility and some major surgery and to give one or two lectures on orthopaedic surgery to the students and staff. Some of these visits are made by steamer (to Barasal) by air or by road, usually with something more robust than the Ford Escort I drive in Dhaka. One visit to a school for disabled children last year involved one and a half hours in a small boat, punting over flooded paddy fields, after the 4-wheel drive Land Rover could go no further; the potholes which form in the roads are tremendous by the end of the Monsoon season, perhaps 10" deep and 2 foot across, remorselessly deepened by the heavy loads and strong wooden wheels of the farmers carts.

When the surgeons have worked for some years, the British Council respond to requests to get them over to the U.K. to enlarge their horizon, or that they may acquire special skills. At an earlier date in the young surgeon's career such visits may lead to frustration on their return to facilities dictated by poverty, which will not have appreciably changed. A full training in a Western country can make a surgeon incapable of accommodating to such difficult conditions. He turns to private practice, where conditions are rather better, or goes abroad, his training lost to his native land.

Great concern has been felt in the Indian subcontinent over the recognition of medical qualifications by the British General Medical Council. Between 1972 and 1983 no qualification in Bangladesh was acceptable so that a visit to Britain could only take place in the capacity of an observer. Visits in 1981 and 1982 from the Royal College of Surgeons of Edinburgh have resulted in a reduction in the annual intake of Medical students into the Dhaka Medical College and an improvement in the teaching facilities. This College, to which medical students have always flocked as their first choice, has been accepted as producing a registerable qualification since the year 1983. There are four Medical Colleges out of the eight in Bangladesh which give an M.B. Dhaka, but only those from the Dhaka Medical College obtain a degree which the British General Medical Council will register.

Let me finally touch on the general pattern of the delivery of medical services to the community in this grossly over-populated nation. The Ministry is called the Ministry of Health and Population Control, accentuating the importance attached to this latter. Contraception is encouraged by doctors and 'motivators'. All doctors are instructed in tubal ligation and the World Banks sole deviation from its strictly financial role is to build a splendid Department for Population Control in each Medical College in the country. Every one recognizes the critical importance, even the husband brining his wife for tubal ligation, rather than he having a vasectomy! As the 50% death rate in childhood falls, the population can only rise without effective contraceptive programmes.

The general pattern of health services has been well conceived but there tends to be much disruption with each change of Government, tending always to the opinion that much must be changed with consequent disruption of progress. Eight Medical College produce *M.B. graduates* and in addition there are colleges which qualify Assistant Medical Officer after a three year training and these latter work in health centres. The radiographers and pathological technicians and others are well trained at the Paramedical School in Dhaka, while other personnel receive their training in the larger Rural Health Centres. Such a centre will use its upper floor for training courses. The 'Birth Attendant' is recruited from the native midwives, is given a 3–4 week course and returned to her work equipped with a midwifery bag and much more up to date knowledge. 'Motivators' of both sexes are trained to go into the villages and explain the benefits of family planning. Tubal ligation is widely practised with a mortality of 2 to 10,000. These operations are undertaken by doctors in the Rural Health Centres or Rural Hospitals, usually under local anaesthesia and deep sedation. (Pethidine and Diazepam by intramuscular and intravenous routes.) 'Sanitory Workers' are trained in the construction of pits and water seal latrines. 'Educators' are a senior grade who will instruct in such matters nearer the village level. 'Voluntary Health Workers' come from Voluntary Health Committees in every village, they are supported by Government funds only while on the course and are never on the payroll. 'Barefoot doctors' are chosen by Government officials in the village, have a nine month course, return to their villages on a salary for a further nine months, after which they rely on fees for their livelihood. 'Oral Hydrologists' are trained and sent out to the villages. This revolution in the treatment of cholera and diarrhoea in children has dramatically changed the whole outlook and these oral hydrologists spread the good news with treatment in the villages.

A description of these training courses outlines the health services at the Village level while at the Rural Health Centre, with its subcentres more elaborate medical services are available – outpatients are held, an accident and emergency service is always available and minor surgery, such as for hernia and hydrocele and tubal ligation is undertaken. Streptomycin and other injections are given and, armed with a deep freeze cabinet, these centres can take their part in the cold chain which has to be set up to get the still active vaccine to the child in the village – the vaccines tend to arrive and depart in Thermos flasks packed with ice. Two pathological technicians provide a pathological service and the 'compounder' dispenses the medicines. There are 5–8 Medical Officers and in some centres Assistant Medical Officers. The former will do private practice in the evenings, sitting in a chemist shop and prescribing for supply on the premises. All Medical Officers have to do one year of Rural Service and this can be a most important year in their experience. Some Health Centres, well led, must provide a great experience, others can leave the young doctor with a feeling of frustration and disillusion; only the Health Administrator with drive will acquire for his Health Centre those essentials which in such a poor country are always in very short supply.

And so we leave this glimpse of a very different world, overfilled with poverty and lack of opportunity, but where there are many struggling towards the creation of a life enjoying more health and human dignity. They require our assistance in giving a market for their products at a fair price and for aid in the form of a fishing rod rather than a fish.

Arthur L. Eyre-Brook

### From Our Foreign Correspondent

### Medical Officer in the TRANSKEI:

'What did you gain from the seven months you spent in Africa?', a consultant asked me at a job interview.

The answers I gave differed somewhat from those I might have thought of if the question had been posed prospectively. I had only a vague notion as to what I, recently qualified and English speaking, could either contribute or achieve when I answered an advertisement in the BMJ for doctors in rural Transkei. On seeing a range of pathology that is now, fortunately, a thing of the past in Britain, it rapidly became apparent that there was plenty to be done by whoever was prepared to do it. Debate as to who should be doing what remains a secondary issue in such a context. Despite initial misgivings over the wisdom of seven months 'out' from the British job scramble (so far, unfounded), I would thoroughly recommend this type of experience to young doctors who are interested in widening their horizons. On the basis of my own experiences, I shall try to convey some idea of the challenges and limitations to be encountered.

Holy Cross, a government hospital in Pondoland (Eastern Transkei), was, in common with all mission hospitals, taken over by the state on assumption of independence by Transkei as a black homeland in 1977 from South Africa. It was a collection of low brick buildings grouped around a beautiful red flowering flame tree. In stark contrast were the surrounding sunbaked clusters of beehive huts, the traditional dwellings of the Pondos. Generations of wood collection for the cooking fires and grazing by goats had largely denuded the hilly landscape.

The Pondos (Figures 1 and 2) were peaceable till roused by dacha (marijuana) or maize beer, when faction fights could erupt. These were often not insubstantial affairs – one fight while I was at Holy Cross resulted in over thirty deaths by stabbing or clubbing with knobkeris (wooden cudgels). For much of the year the men were absent working as migrant labourers in the Natal sugar plantations and Transvaal mines in order to support their families. The womenfolk were left to cope with agricultural and domestic demands. The maize fields they tended were parched following the failure of the vital annual rains and so the hospital was having to deal with large numbers of malnourished and diseased children.

**Figure 1**

Pondo villagers.

**Figure 2**

Village beauty.

*Hospital work*

The hospital was staffed by Transkeian trained nurses – indeed, Holy Cross was a nurse training school – and by five expatriate doctors, responsible for over three hundred inpatients as well as out-patients. Our guiding light was a British trained Transkeian surgeon who imparted to us both clinical skills and his local knowledge. We looked forward to his weekly visit, not only for the help we received but also for the convivial dinner we had together after the work was finished, and the showing of the film he hired for us.

The distinctive clicking sounds of the Xhosa language, spoken in Transkei, drew attention to the long queue outside outpatients which assembled daily. This was where the greater part of the hospital's work was done. There I learned rapidly to use my eyes, ears and hands to diagnose and treat. Modern diagnostic tests were neither available nor often needed in a country where disease presented so late and so floridly. Confidence must rest with clinical medicine when there is little to compete with it. Basic X-ray and laboratory facilities were, however, used to the full when tackling such common problems as differentiating active from inactive tuberculosis and diagnosing syphilis and parasitic infections. I soon became accustomed to the local problems and treatments available from our well stocked pharmacy.

Patients needing admission to the wards could always be accommodated either in beds or, if needs be, on floor mattresses. The nurses, who had been trained in English, acted as translators when necessary, as it usually was. The general men's ward, which I looked after, catered for a wide variety of problems: in particular trauma, often from agricultural accidents, and the diagnosis of tuberculosis. Patients with tuberculosis, be it pulmonary, meningitis, spinal, arthritis, pericarditis or generalised, were transferred to a specialised ward when well enough. Three months inpatient treatment was followed by eighteen months outpatient therapy. As in all conditions drug compliance was a problem in the effective control of the disease, both individually and throughout the community.

The adage of 'see one, do one, teach one' is often but not always possible in an understaffed hospital two hundred kilometres along a bumpy untarred road from the nearest referral centre. Soon after I arrived, when all but two doctors were away for the weekend, a multigravid women who had been labouring at home presented with a uterus ruptured across a previous caesarian section scar. At laparotomy it became apparent that the only possible treatment was a hysterectomy, which was duly done. The result was successful only because all the doctors had to either have or acquire rapidly basic obstetric and anaesthetic skills. Any combination of two doctors on duty could thus cope with any surgical emergency.

Having done no obstetrics other than as a student, I was sent for five days to the obstetric unit in Umtata, the capital, to learn how to assess women in labour and to do caesarian sections. The decision to intervene in labour, never being an easy one, was made more difficult in a society where inability to bear children is seen as an abject failure and operative delivery as partial failure. We considered caesarian section the greater of the two evils (not intervening being the other) in cases of fetal distress, due to subsequent high perinatal mortality and future risk of ruptured uterus, for many women would refuse to come into hospital again after an operative delivery. Inevitably the number of stillbirths reflected this policy. Cephalopelvic disproportion was thus the main indication for caesarian section.

It is inconceivable that a women in Britain could lie in obstructed labour for three days. The unfortunate women who I saw in this predicament was, moreover, the daughter of a midwife, and suffered recto- and vesico-vaginal fistulae as well as bilateral sciatic nerve palsies. For me, the challenge of managing women in labour was in many ways as constructive as the operative skills I gained.

It is easy to fall into bad habits when working in isolated circumstances, but supervision of the surgical work helped to discourage these. Regular theatre lists always included skin grafting of burns, particularly among untreated epileptics who fell into open cooking fires during fits, as well as the surgery of other trauma and infections. Cases of acute osteomyelitis were drilled as emergencies,.but we also had to deal with the chronic sequelae. In addition to the main theatre there was a smaller one in outpatients which was in daily use for the setting of fractures and draining of abscesses. Surgical specimens were sent to Durban but there was a three week wait for the histology reports, and so patients would often be started on anti-tuberculous therapy while awaiting the histological diagnosis from a lymph node or synovial biopsy.

Elective medical students were a great help in giving anaesthetics. We all relied heavily on short acting intravenous agents, though often I had to induce anaesthesia, scrub up and operate on the same patient, leaving a nurse to hold the mask. It was, however, a firm rule that during intubational anaesthesia, which we used for abdominal operations including caesarian sections, there were always two doctors in theatre.

*Primary health care*

Diagnosing and treating patients in hospital is relatively easy by comparison with attempting to influence aetiological factors. Primary health care has a low priority as the treatment of disease is perceived by the consumers of health services as more important than its prevention. In common with other third world countries, many local graduates in Transkei preferred to practise in towns, and attempts to provide health care on a local basis met with only limited success. Teams of nurses regularly went out to the villages, but the effort was small in comparison with what was needed.

Cultural factors play a significant role in the acceptance or otherwise of health education from expatriate doctors and nurses trained in a system foreign to the villages. Many a time, despite great efforts to persuade mothers to breast feed their babies, they were seen clutching bottle and teat a few weeks after leaving hospital. The reason given was that the husband's mother, who supervised domestic affairs, insisted that bottle feeding was more 'advanced' than the breast.

Probably the greatest help to all health workers in assessing and improving the condition of young children was the 'Road to health' card, which was kept by the mother. Immunisations and illnesses were recorded and weight-for-age graphically detailed with the third and fiftieth centiles shown. The cards were used in addition to clinical examination to identify children with poor weight gain and frank malnutrition. There were many cases of both marasmus and kwashiorkor, and some showing features of both.

*Meeting local aspirations*

Expectation is of a short life, punctuated by the birth of many children, some of whom will die. Despite this, death is grieved over no less than in Britain but probably accepted with greater equanimity. Nonetheless, good health and in particular fertility are much sought after. I saw a local chief who complained that his wife had borne no children, a matter of concern where the lobela (the dowry the husband pays to his wife's family) may be returned if the marriage is childless. Examination of his semen revealed azoospermia, and despite indicating that the problem might be his, he was most insistent that his wife be sent to Umtata and treated for infertility. It was only with great difficulty that these demands were resisted.

The medical profession did not have the same monopoly as in the NHS, but met with fierce competition from the witch doctors who, despite higher charges, attracted excellent business. Daily, patients were seen with scarification marks over their wounds following visits to witch doctors and traditional village healers. Severe consequences could result, such as the compounding of closed fractures. The local community recognised those conditions amenable to treatment by Western medicine and those less so. It was rare to see psychiatric patients as they were taken to the witch doctors who, no doubt, were more adept at differentiating the influences of excessive dacha smoking from those of evil spirits. Sadly, we could do little for the chronically disabled, and this reality was accepted.

(The author was a Medical Officer at Holy Cross Hospital, Transkei, in 1983.)

Andrew Farkas

## Figures and Tables

**Figure 1 f1:**
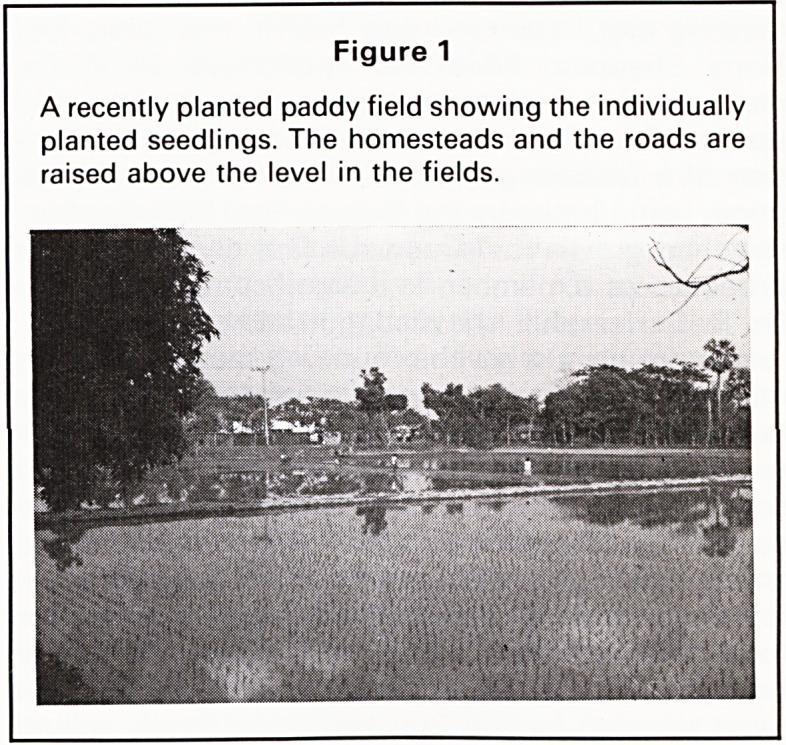


**Figure 2 f2:**
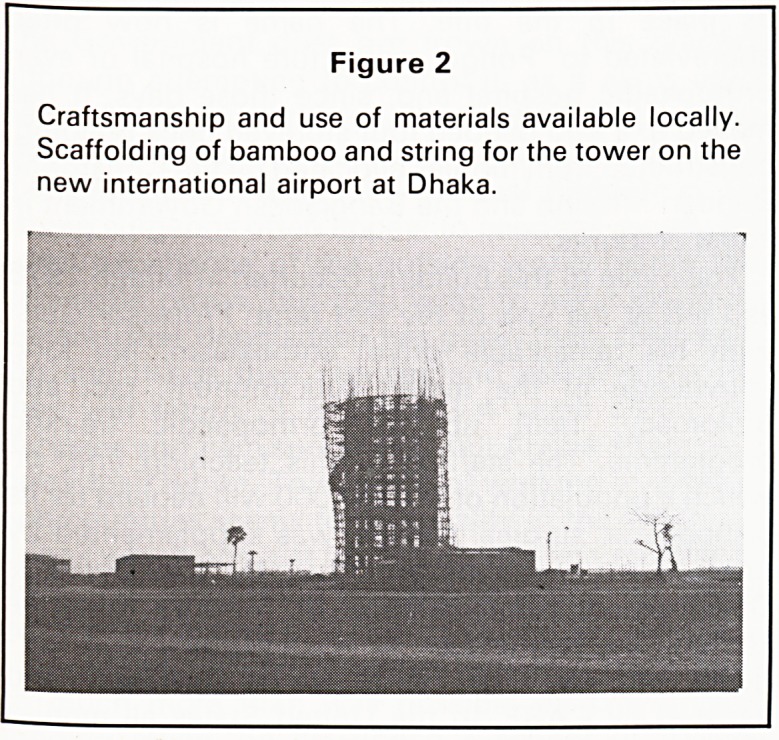


**Figure 3 f3:**
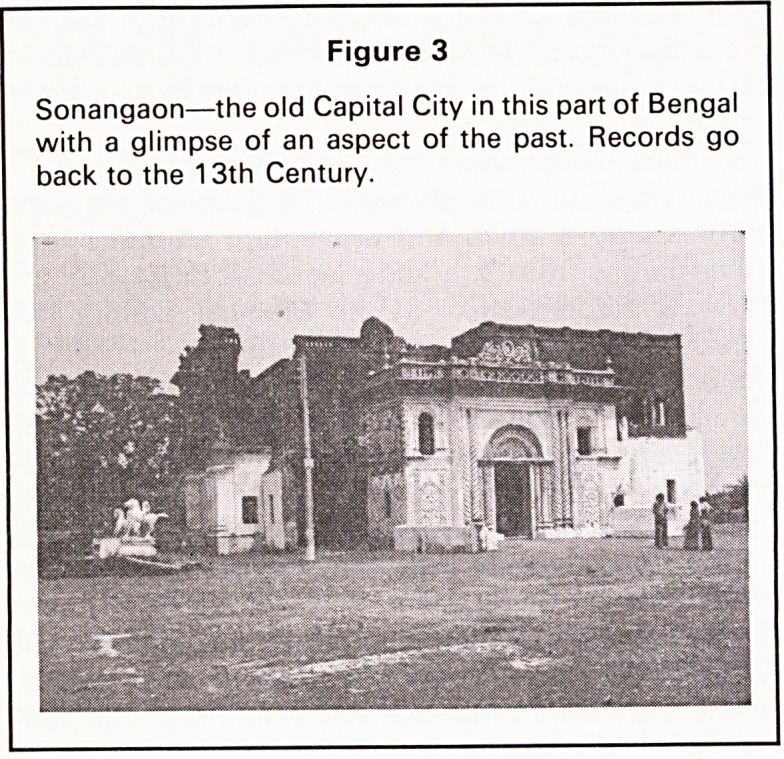


**Figure 1 f4:**
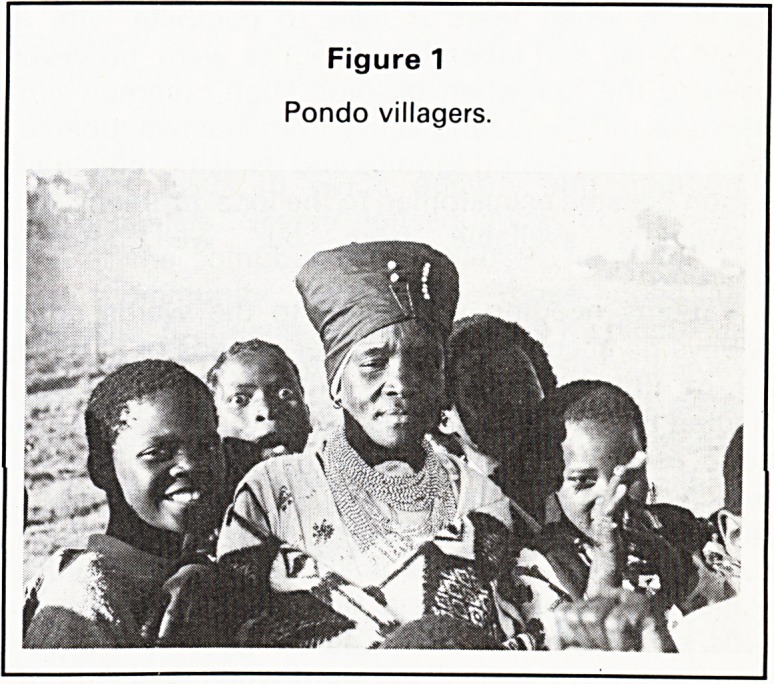


**Figure 2 f5:**